# Complete fulfillment of expectations is associated with greater satisfaction after shoulder arthroplasty: results from a prospective multicenter cohort study

**DOI:** 10.1016/j.jseint.2026.101651

**Published:** 2026-01-29

**Authors:** Brechtje Hesseling, Youri Berends, Sophie van der Linden, Jaap J. Tolk, Just A. van der Linde, Cornelis P.J. Visser, Karin Slot, Denise Eygendaal, Barbara A.M. Snoeker, Nina M.C. Mathijssen

**Affiliations:** a Reinier Haga Orthopedic Center, Zoetermeer, The Netherlands; b Department of Orthopaedics and Sports Medicine, Erasmus MC, University Medical Center Rotterdam, Rotterdam, The Netherlands; c Department of Medical Psychology and Psychiatry, Reinier de Graaf Hospital, Delft, The Netherlands; d Department of Orthopaedics and Sports Medicine, Erasmus MC – Sophia Children's Hospital, University Medical Center Rotterdam, Rotterdam, The Netherlands; e Department of Orthopaedics, Alrijne Hospital, Leiderdorp, The Netherlands; f Centre for Orthopedic Research Alkmaar (CORAL), Department of Orthopaedic Surgery, NorthWest Clinics, Alkmaar, The Netherlands; g Department of Epidemiology and Data Science, University Medical Center, Amsterdam, The Netherlands; h University of Amsterdam, Amsterdam, The Netherlands; i Department of Clinical Epidemiology and Orthopaedics, Lund University, Lund, Sweden

**Keywords:** Shoulder arthroplasty, Pre-operative expectations, Post-operative expectation fulfillment, Probabilistic expectations, Value-based expectations, Post-operative satisfaction

## Abstract

**Background:**

With this study, we aim to explore pre-operative probabilistic expectations (what does the patient realistically expect) and value-based expectations (what does a patient consider important), post-operative expectation fulfillment, and the relationship with post-operative satisfaction in shoulder arthroplasty (SA) patients. We hypothesized that higher expectation fulfillment is related to higher post-operative satisfaction, corrected for baseline level of optimism.

**Methods:**

In this prospective multicenter cohort study, 230 SA patients were included. These patients completed pre-operative questionnaires measuring probabilistic (Sunnybrook questionnaire) and value-based expectations (Hospital for Special Surgery Shoulder Expectations questionnaire) as well as optimism (Life Orientation Test – Revised). Post-operatively, expectation fulfillment/improvement and post-operative satisfaction were measured. We used ordinal logistic regression to study whether expectation fulfillment is related to post-operative satisfaction, corrected for baseline level of optimism.

**Results:**

For pre-operative probabilistic expectations, patients expected improvement on a median of 5 domains (interquartile range 4-6) out of 6 possible domains. For pre-operative value-based expectations, patients had a median of 5 (interquartile range 3-9) out of 17 possible expectations that they valued as ‘Very important’. At 6 months and 12 months, the median percentage of completely fulfilled probabilistic expectations per patient was 75% and 80%, respectively. The median percentage of completely improved ‘very important’ value-based expectations per patient was 7.7% and 14.3%. Patients with higher percentages of fulfilled or improved expectations had higher odds of being ‘very satisfied’ post-operatively.

**Conclusion:**

This study shows that fulfillment of probabilistic expectations and improvement on value-based expectations is associated with post-operative satisfaction after SA.

In the past years, joint arthroplasty research has shifted its focus from mainly biomedical factors to more psychological factors, among which pre-operative expectations and expectations fulfillment are included. In the literature, pre-operative expectations can be measured with 2 different main paradigms: probabilistic (what a patient realistically expects) and value-based (what is important to a patient).^7^ Hafkamp et al^4^ found in their systematic review that higher expectation fulfillment was linked to higher post-operative satisfaction for total knee arthroplasty (TKA) and total hip arthroplasty (THA). For the level of pre-operative expectations (as opposed to expectation fulfillment) they found conflicting evidence. The studies in this review used both paradigms to measure expectations and the review did not discriminate between these 2.

Regarding the influence of expectations in shoulder arthroplasty (SA) patients the literature is scarcer. A recent systematic review on pre-operative expectations by Claes et al^1^ could only include 4 studies. They concluded that there is moderate evidence for no association between pre-operative expectations and post-operative outcomes. Moreover, only 1 study has investigated expectation fulfillment in SA patients^3^ and found weak correlations with satisfaction (*r* = 0.17-0.37) but did not quantify the magnitude of the relationship. Also, they only studied probabilistic and not value-based expectations.

To prepare patients well and to enable surgeons to align their expectations to those of their patients, we need more insight into the association between expectation fulfillment and post-operative satisfaction in this population. Furthermore, we need to understand if it matters with which paradigm (probabilistic or value-based) and corresponding instrument expectations are measured to provide patients with correct information. To date, we have not found any study that measured both paradigms at the same time in a single group of patients. In this study, we therefore set out to answer the following three research questions: (1) pre-operatively, what do SA patients expect (probabilistic expectations) and what is important to SA patients (value-based expectations)? (2) What percentage of pre-operative expectations becomes fulfilled at 6 and 12 months after SA? and (3) Do patients with higher expectation fulfillment have higher post-operative satisfaction scores? We hypothesized that higher expectation fulfillment is related to higher post-operative satisfaction, corrected for baseline level of optimism.

## Materials and methods

### Study design and setting

For this multicenter, prospective cohort study patients were recruited between April 2018 and September 2021 from 4 teaching and/or general hospitals in the Netherlands. All participating surgeons were fellowship-trained high volume shoulder surgeons. Patients were followed for 1 year and study data were collected from the electronic patient records, through a paper diary and through surveys (either through email or on paper).

### Participants

We screened all patients indicated for SA consecutively for study eligibility. Patients were eligible for inclusion when they were 18 years or older, scheduled to undergo primary or revision SA (either hemiarthroplasty, anatomic total shoulder arthroplasty [aTSA], or reverse total shoulder arthroplasty [rTSA]), able and willing to participate, and had good understanding of the written and spoken Dutch language. Patients were excluded for cognitive impairment and arthroplasty for acute fractures. All participating patients provided written informed consent prior to study participation.

### Description of treatment

Each surgeon typically used the deltopectoral approach and general anesthesia with interscalene block. Patients were kept overnight and were generally discharged the next day. They were instructed to use a sling for six weeks and to only perform passive exercises during the first two weeks. After 2 weeks, patients could increase their exercises to include active-assisted and subsequently active exercises.

### Variables, outcome measures, data sources, and bias

#### Data collection

Patients completed questionnaires at baseline and at 6 and 12 months post-operatively; this was part of a larger study in which patients also completed a daily diary during the early recovery period. The questionnaires contained questions on general patient characteristics, pain, function, quality of life, psychological factors, expectations, and (post-operatively only) satisfaction. For the research questions in this manuscript, only questionnaires for patient characteristics, optimism, expectations, and satisfaction were used. Results on early recovery using the diary data were published previously in this journal.^6^

#### Optimism

The validated Life Orientation Test – Revised^15^ was used to measure pre-operative generalized optimism. It is a validated 10-item questionnaire answered on a 5-point Likert scale ranging from 0 (strongly disagree) to 4 (strongly agree). The total score thus ranges from 0 (low optimism) to 24 (high optimism).

#### Expectations

Probabilistic expectations were measured pre-operatively and post-operatively with the Sunnybrook expectation questionnaire.^14^ This questionnaire contains 6 domains (ie, pain, range of motion [ROM], ability to perform activities of daily living, ability to interact with and take care of others, ability to return to previous leisure/recreational/sports activities, and potential to achieve full recovery following surgery; domains written out in figures in Results section) and asks whether patients pre-operatively expect improvement on these domains and if so, how much improvement.

For post-operative expectation fulfillment, the phrasing of the questions was changed from, for example, ‘Do you expect pain relief from the surgery’ to ‘Did the surgery reduce your pain?’ in a modified Sunnybrook questionnaire.

Value-based expectations were measured pre- and post-operatively with the Hospital for Special Surgery (HSS) Shoulder Expectations questionnaire.^10^ This questionnaire contains 17 items (similar to the domains of the Sunnybrook questionnaire but in more detail, domains written out in figures in Results section) and asks patients to rate the importance of each expectation.

In this value-based questionnaire, patients reported how important each item was but not if they actually expected improvement on this item after surgery. Therefore, fulfillment of these expectations could not be assessed; instead, we measured post-operative improvement. We reworded the instruction for the modified HSS questionnaire from ‘How important are these expectations in the treatment for your shoulder?’ to ‘For these expectations, which answer fits your situation best?’. Answer categories were on an 8-point Likert scale ranging from ‘complete improvement’ to ‘much deterioration’.

Both the pre-operative and modified (post-operative) questionnaires can be found in the Supplementary file (Tables S1-S4).

Since no validated Dutch translations were available for both questionnaires at the start of the study, self-translated versions were used.

#### Satisfaction

We measured post-operative satisfaction with a single question (‘How satisfied are you up to now with the results of your surgery?’). The answers were on a 5-point Likert scale ranging from ‘very satisfied’ to ‘very dissatisfied.’

### Ethical approval

METC Leiden, Den Haag, Delft (formerly METC Southwest Holland) in the Netherlands decided that the study did not fall under the scope of the Medical Research Involving Human Subjects Act because of the minimal patient burden (METC-no. 17-117). The study was conducted to conform the Declaration of Helsinki and the European Medicines Agency Guidelines for Good Clinical Practice.

### Statistical analysis, study size

We described characteristics of the entire sample with number and frequency for categorical variables, with mean and standard deviation for normally distributed continuous data and with median and interquartile range (IQR) for non-normally distributed data.

All statistical analyses were performed in R (version 4.4.1)^12^ with RStudio (Posit, Boston, MA, USA).^11^ As described in our previous publication, the sample size of 230 subjects was based on the analysis of the early recovery results and on the feasibility of including the number of subjects. Missing data were not imputed.

The statistical analyses per research question are described below.

#### Pre-operative expectations (research question 1)

To describe the pre-operative expectations, our analysis for both types of expectations was two-fold: on the level of each expectation and on the patient level.

First, for each separate expectation, we calculated the percentages of patients within each answer category.

Next, for each patient, the number of probabilistic (Sunnybrook) expectations that were actually present per patient was calculated (eg, the number of expectations scored with any of the ‘yes’ categories). Similarly, the number of value-based (HSS) expectations that were rated as ‘very important’ per patient was calculated.

#### Expectation fulfillment and improvement (research question 2)

For probabilistic expectations, expectation fulfillment was categorized as ‘completely fulfilled,’ ‘somewhat fulfilled,’ or ‘not at all fulfilled.’

For value-based expectations, we collapsed our 8-point Likert scale into similar categories ‘completely improved,’ ‘somewhat improved,’ or ‘not at all improved.’

Similarly to question 1, the analysis was done on the expectation level and on the patient level.

First, on the expectation level, we calculated for each probabilistic and value-based expectation the percentages of patients rating that expectation as ‘completely fulfilled’ and as ‘completely or somewhat fulfilled’ (probabilistic) or ‘completely improved’ and ‘completely or somewhat improved’ (value-based), at both time points.

Next, on the patient level, we calculated for each patient the percentage of their pre-operative expectations that were ‘completely fulfilled/improved’ and ‘completely or somewhat fulfilled/improved,’ also at both time points.

Patients that did not complete the pre-operative questionnaires were excluded from these analyses. For more details on this analysis, see Supplementary file.

#### Relationship of expectation fulfillment/improvement with post-operative satisfaction (research question 3)

To account for the ordinal nature of our satisfaction measure, we performed ordinal logistic regression analyses using the ‘MASS’ package.^18^ In these models, the dependent variable was post-operative satisfaction and the focal predictor variable was the percentage probabilistic expectation fulfillment or percentage value-based expectation improvement. The percentages were first divided by 10 so that the odds ratios represent a step of 10% increase in the predictor variable.

For ease of interpretation and due to small numbers of not satisfied/not dissatisfied and unsatisfied patients, the outcome categories ‘not satisfied/satisfied,’ and ‘very dissatisfied’ were merged into a single category ‘not satisfied.’

For each type of expectations (ie, probabilistic and value-based), we ran 4 separate models, as follows:

- Focal predictor ‘complete fulfillment/improvement’ at six months
- Focal predictor ‘complete fulfillment/improvement’ at twelve months
- Focal predictor ‘complete or some fulfillment/improvement’ at six months
- Focal predictor ‘complete or some fulfillment/improvement’ at twelve months

Within each type of expectation we used the Bonferroni method to correct for multiple testing and set our *P* value threshold at *P* = .0125. Patients that did not complete the pre-operative questionnaires were excluded from these analyses.

The degree of pre-operative generalized optimism was accounted for by adjusting for baseline Life Orientation Test – Revised scores in each model. Given the common rule of thumb to only add 1 variable per 10 cases, adding more variables to the model was not considered prudent: at 6 and 12 months, only 21 and 19 patients were ‘not satisfied,’ respectively.

No multicollinearity was detected for any model. We used the Brant-Wald test to assess for each model whether the proportional hazard assumption held and the Lipsitz goodness of fit test to investigate whether the model fit our data well. Two models at 12 months proved to be problematic; the Supplementary file details how these problems were handled.

Finally, to aid in the interpretation of the models, we calculated and visualized the predicted probabilities for all different levels of the predictor variable.

## Results

**Figure 1:**
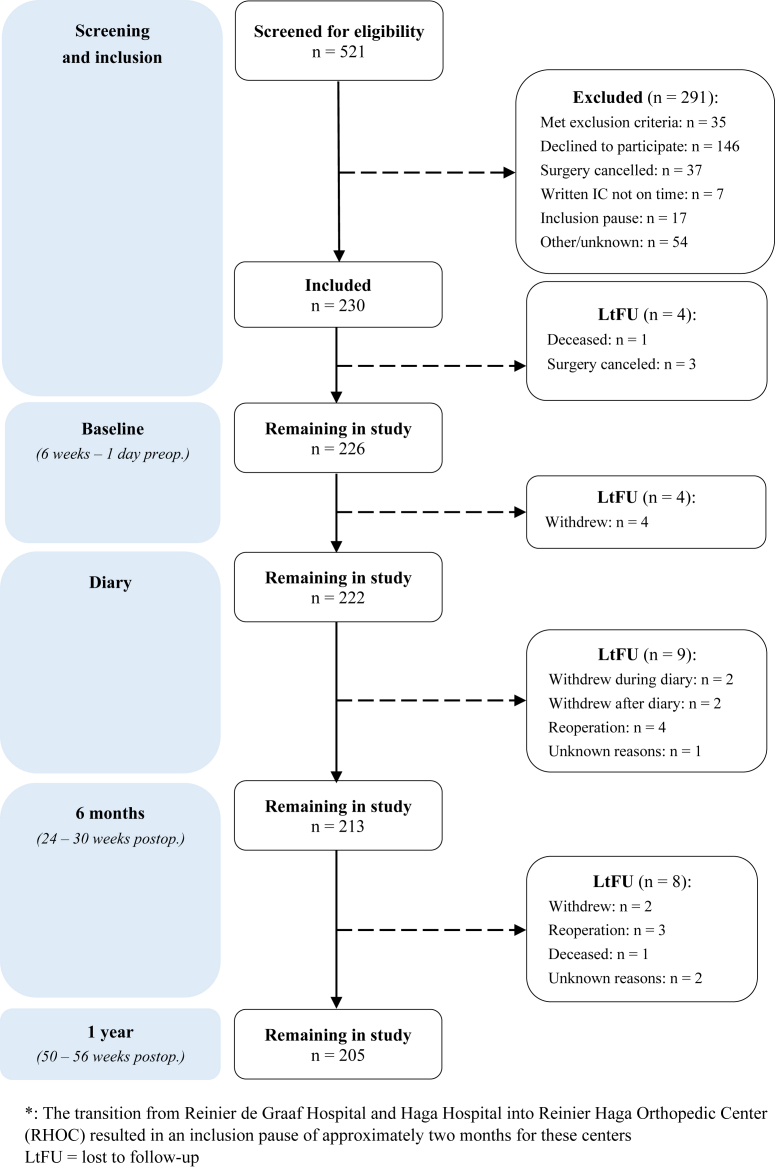
Study flow chart.

Fig. 1 shows the flow of patients throughout the study. We included 230 patients with a mean age of 69.9 (standard deviation 8.5) years, of which 160 (69.6%) were female. The most common diagnosis was osteoarthritis (60.4%) and the most frequently used type of implant was rTSA (66.1%). For complete patient characteristics, see Table I.

**Table I:** Descriptive statistics of the study sample.

Variable	Entire sample (N = 230)
Demographic	
Age (mean (SD))	69.9 (8.5)
Sex (no. (%))	
Male	67 (29.1%)
Female	160 (69.6%)
ASA (no. (%))	
Class I	22 (9.6%)
Class II	138 (60.0%)
Class III or higher	65 (28.3%)
BMI (no. (%))	
Normal weight	61 (26.5%)
Overweight (BMI 25-30)	94 (40.9%)
Obese (BMI ≥30)	70 (30.4%)
Duration of complaints in years (median [IQR])	3.0 [1.5-6.0]
Indication (no. (%))	
OA	139 (60.4%)
mRCT/CTA	51 (22.2%)
Other	35 (15.2%)
Cultural background (no. (%))	
Dutch	212 (92.2%)
Surinamese	2 (0.9%)
Other	7 (3.0%)
Religion (no. (%))	
Christian	121 (56.3%)
Catholic	4 (1.9%)
Jewish	1 (0.5%)
Other	5 (2.3%)
Not religious	66 (30.7%)
I'd rather not say	7 (3.3%)
Surgical	
Primary/revision (no. (%))	
Primary	220 (95.7%)
Revision	5 (2.2%)
Type of prosthesis (no. (%))	
aTSA	64 (27.8%)
rTSA	152 (66.1%)
HA	7 (3.0%)
Dominant side (no. (%))	
Yes	100 (43.5%)
No	110 (47.8%)
Baseline psychological factors	
LOT-R optimism subscale (mean (SD))	8.1 (1.9)

*ASA*, American Society of Anesthesiologists; *BMI*, body mass index; *OA*, osteoarthritis; *mRCT*, massive rotator cuff tear; *CTA*, cuff tear arthropathy; *aTSA*, anatomic total shoulder arthroplasty; *rTSA*, reverse total shoulder arthroplasty; *HA*, hemiarthroplasty; *LOT-R*, Life Orientation Test - Revised; *SD*, standard deviation.

Of the 230 included patients, 205 patients completed the 1 year follow-up period; 11% of patients were lost to follow-up.

### Pre-operatively, what do SA patients expect (probabilistic expectations) and what is important to SA patients (value-based expectations)?

**Figure 2:**
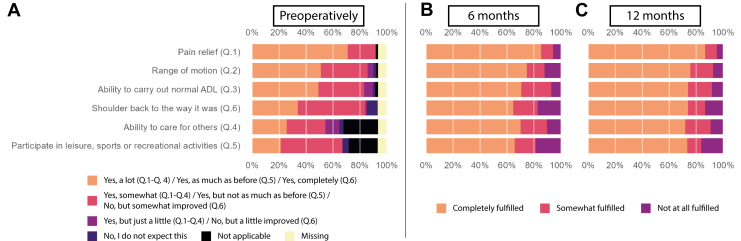
(**A-C**) Barplots showing for probabilistic expectations (Sunnybrook). (**A**) How many patients responded to each of the answer categories pre-operatively, and (for patients who had that expectation pre-operatively) the degree of fulfillment at (**B**) 6 months and (**C**) 12 months.

**Figure 3:**
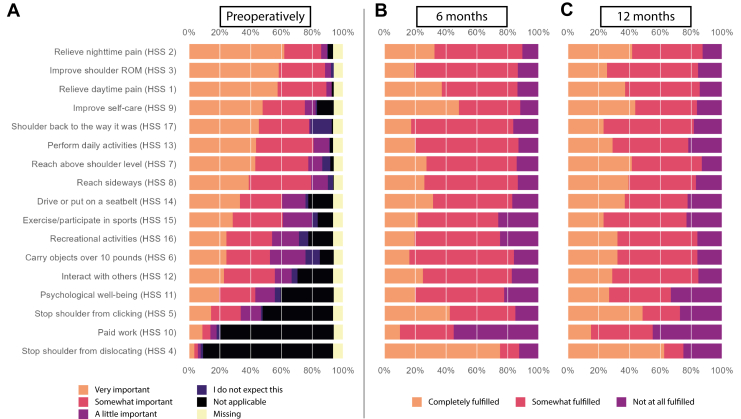
(**A-C**) Barplots showing for value-based expectations (HSS) (**A**) how many patients responded to each of the answer categories pre-operatively, and (for patients who rated that expectation pre-operatively as ‘very important’) the degree of improvement at (**B**) 6 months and (**C**) 12 months.

For pre-operative probabilistic expectations, patients expected improvement on 5 (median, IQR 4-6) out of the 6 possible domains. For pre-operative value-based expectations, patients had 5 (median, IQR 3-9) out of the 17 possible expectations that they valued as ‘Very important’. Pain relief and improvement in ROM were both most often expected (probabilistic) and most often rated as ‘very important’ (value-based). Figs. 2*A* and 3*A* show the distribution of answers for the Sunnybrook questionnaire and the HSS, respectively. Exact numbers and percentages can be found in Table S5 and S6 in the Supplementary File.

### What percentage of pre-operative expectations becomes fulfilled at 6 and 12 months after shoulder arthroplasty?

A higher percentage of probabilistic expectations were fulfilled than value-based expectations were improved at both time points. At 6 months and 12 months, the median percentage of completely fulfilled probabilistic expectations per patient was 75% and 80%, respectively (IQR 33.3%-100% for both time points). The median percentage of completely improved ‘very important’ value-based expectations per patient was 7.7% (IQR 0%-50%) and 14.3% (IQR 0%-57.1%), at 6 and 12 months, respectively. Figs. 2, *B* and *C* and 3, *B* and *C* show the percentages ‘completely fulfilled/improved,’ ‘somewhat fulfilled/improved,’ and ‘not at all fulfilled/improved’ at both time points for the Sunnybrook questionnaire and HSS, respectively. Tables S7 and S8 in the Supplementary File show exact numbers and percentages.

### Do patients with higher expectation fulfillment have higher post-operative satisfaction scores?

Patients with higher percentages of fulfilled or improved expectations had higher odds of being ‘very satisfied’ post-operatively. At 6 and 12 months, 50.4% (116 of 230 patients) and 53.9% (124 of 230 patients) were ‘very satisfied,’ 30.4% (70 patients) and 24.8% (57 patients) were ‘satisfied,’ 9.1% (21 patients) and 8.3% (19 patients) were ‘not satisfied.’ Ten percent (23 patients) and 13% (30 patients) had missing satisfaction scores. Table II displays the odds ratios, confidence intervals and corresponding *P* values for all models, as well as model diagnostics. The model for ‘completely fulfilled’ probabilistic expectations at 12 months was not reliable and is therefore not reported in Table II (see Supplementary file for details).

**Table II:** Model estimates and diagnostics for all ordinal logistic regression models.

Model	OR∗ (95% CI)	*P* value	*P* value Lipsitz	*P* value Brant-Wald
Probabilistic expectations (Sunnybrook)				
6 mo				
Model 1			.32	.19
Per 10% completely fulfilled	1.44 (1.30-1.59)	<.001		.07
Baseline optimism	0.98 (0.90-1.07)	.63		.77
Model 2			.53	.45
Per 10% completely/somewhat fulfilled	1.49 (1.32-1.68)	<.001		.23
Baseline optimism	0.99 (0.91-1.07)	.78		.58
12 mo				
Model 3			.02	.88
Per 10% completely fulfilled	-†			.97
Baseline optimism	-†			.62
Model 4			.52	.89
Per 10% completely/somewhat fulfilled	1.25 (1.12-1.39)	<.001		.69
Baseline optimism	1.08 (0.98-1.19)	.10		.78
Value-based expectations (HSS)				
6 mo				
Model 5			.51	.40
Per 10% completely improved	1.66 (1.42-1.93)	<.001		.18
Baseline optimism	.91 (.83-1.01)	.07		.84
Model 6			.27	.51
Per 10% completely/somewhat improved	1.59 (1.37-1.84)	<.001		.39
Baseline optimism	1.01 (.92-1.10)	.89		.39
12 mo				
Model 7			.19	.33
Per 10% completely improved	1.53 (1.32-1.78)	<.001		.18
Baseline optimism	1.04 (0.94-1.15)	.48		.43
Model 8			.41	.19
Per 10% completely/somewhat improved	1.24 (1.11-1.38)	<.001		.08
Baseline optimism	1.10 (1.00-1.21)	.05		.78

∗ OR, odds ratio for moving 1 step upward in satisfaction (ie, ‘Not satisfied’ → ‘Satisfied’ or ‘Satisfied’ → ‘Very satisfied’).

† This model was not reliable.

**Figure 4:**
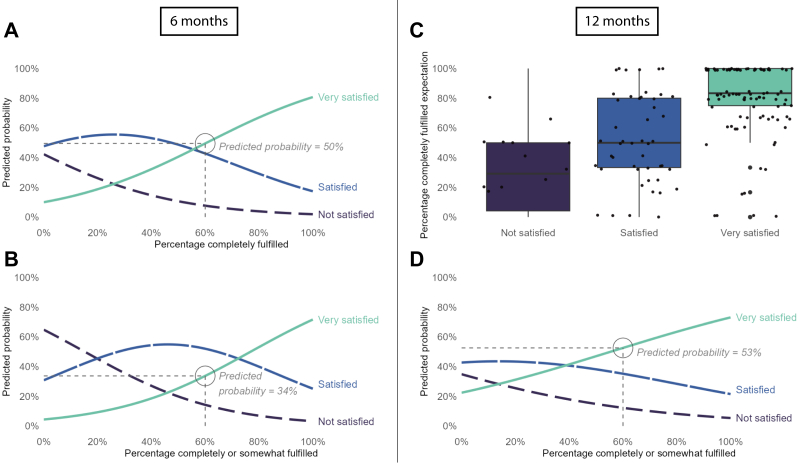
(**A-D**) Plots showing the predicted probabilities of being ‘Very satisfied,’ ‘Satisfied,’ or ‘Not satisfied’ for probabilistic expectations (Sunnybrook), (**A**) for percentage ‘completely fulfilled’ expectations at 6 months, (**B**) for percentage ‘completely or somewhat fulfilled’ at 6 months, (**C**) median and IQR of percentage ‘completely fulfilled’ expectations per satisfaction level at 12 months (this model was unreliable), and (**D**) for percentage ‘completely or somewhat fulfilled’ at 12 months. The 60% fulfilled in the annotations was arbitrarily chosen to support interpretation of the plots.

**Figure 5:**
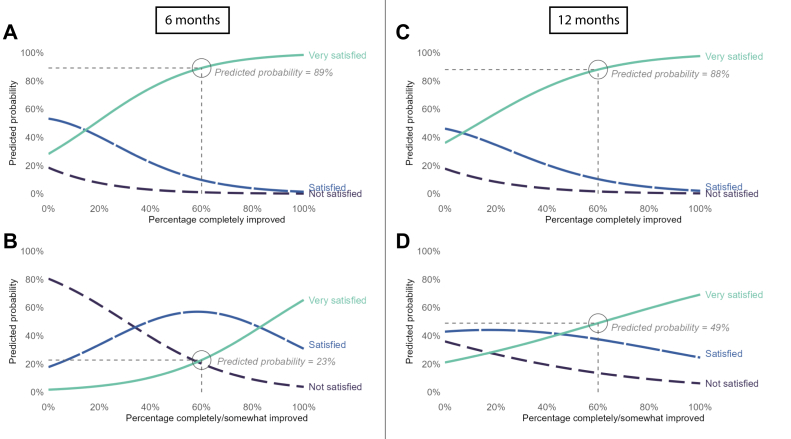
(**A-D**) Plots showing the predicted probabilities of being ‘Very satisfied,’ ‘Satisfied,’ or ‘Not satisfied’ for value-based expectations (HSS), (**A**) for percentage ‘completely improved’ expectations at 6 months, (**B**) for percentage ‘completely or somewhat improved’ at 6 months, (**C**) for percentage ‘completely improved’ expectations at 12 months, and (**D**) for percentage ‘completely or somewhat improved’ at 12 months. The 60% fulfilled in the annotations was arbitrarily chosen to support interpretation of the plots.

To aid in the interpretation of the different models, Figures 4 and 5 visualize the predicted probabilities of the reliable models. These plots show for each level of percentage expectation fulfillment/improvement what the probability is for a patient to be ‘very satisfied,’ ‘satisfied,’ and ‘not satisfied.’ For example, for patients who have 60% of their very important value-based expectations *completely* improved at six months, the predicted probability of being ‘very satisfied’ is 89% (Fig. 5*A*). In contrast, when a patient has 60% of their expectations *completely or somewhat* improved, the predicted probability is only 23% (Fig. 5*B*).

Regarding the qualitative examination of the model for probabilistic expectations at 12 months, Fig. 4*C* shows that the median percentages ‘completely fulfilled’ probabilistic expectations at 12 months for ‘very satisfied,’ ‘satisified,’ and ‘not satisfied’ correspond to the conclusion of the other models. In addition, Figures S1 and S2 in the Supplementary file show the distributions of all focal predictors at both time points split into the 3 satisfaction levels.

## Discussion

This is the first study to measure both probabilistic and value-based expectations within a single joint arthroplasty sample. We found that higher percentages of expectation fulfillment or expectation improvement were associated with higher levels of satisfaction. The predicted probabilities for being ‘very satisfied’ were higher for ‘completely improved’ value-based expectations than for ‘completely or somewhat’ improved value-based expectations.

In TKA and THA patients, expectation fulfillment has been consistently linked to better post-operative outcomes, but for SA patients the literature has been sparse. A better understanding of the relationship between expectation fulfillment and post-operative satisfaction in SA patients and of possible differences between the probabilistic and value-based paradigms will enable the orthopedic surgeon to adequately manage their patients' expectations.

### Pre-operatively, what do SA patients expect (probabilistic expectations) and what is important to SA patients (value-based expectations)

In this study, SA patients pre-operatively ranked pain relief most often as very important, which corresponds to results from other studies that focused on SA and used the value-based HSS questionnaire.^5^^,^^9^^,^^13^ However, exact percentages varied between these studies: daytime pain relief was rated as very important by 86% of patients in the study by Henn et al^5^ and 85% in the study by Lizzio et al,^9^ and by 48% in the study by Rauck et al^13^ and 57% in our study. Improvement in ROM and improvement in self-care were also different between the studies, ranging from 35% to 89% for ROM and from 48% to 82% for self-care. The reason for these differences is not yet clear and could be multifactorial, for example, due to type of arthroplasty or whether expectation fulfillment was determined through written or verbal questioning. Perhaps different methods of expectation management in different hospitals could affect how patients rate expectations, although it remains questionable if value-based expectations can be influenced by pre-operative counseling to the same extent as probabilistic expectations.^16^^,^^17^ After all, knowing what is realistic may not change what is important to a patient.

### What percentage of pre-operative expectations becomes fulfilled at 6 and 12 months after shoulder arthroplasty?

Until this study, only Green et al^3^ investigated expectation fulfillment in aTSA patients, by asking to which degree pre-operative probabilistic expectations ([measured with] the Musculoskeletal Outcomes Data Evaluation and Management Scale [MODEMS]) had been met at a follow-up of two years. They report that expectations were met for a high percentage of patients. The highest level of met expectations was for relief from symptoms and the lowest level for the ability to exercise and do recreational activities. These results show the same tendency as our results on fulfillment of probabilistic expectations at 6 and 12 months.

For value-based expectations, the 7.7% of ‘completely improved’ expectations in our results seems very low. However, we believe this low percentage can be explained by the definitions we employed in our methods. Firstly, the percentage was only calculated on expectations that patients rated pre-operatively as ‘very important’; therefore, improvement on expectations that were of some importance but not ‘very important’ was not included in this calculation. Secondly, ‘complete improvement’ is a high bar to set and perhaps not realistic for all expectations. Large (yet not large enough to rate as ‘complete’) improvements are again not included in the percentage of ‘completely improved’ expectations. Fig. 3*B* and *C* show that for each expectation, the large majority of patients reached either ‘completely improved’ or ‘somewhat improved.’ Fewer patients indicated ‘not at all improved.’

To our knowledge, there are no studies that examined improvement or fulfillment of value-based expectations in SA patients. Our results therefore provide valuable input for surgeons and other health-care professionals to use in their patient counseling; they may add another dimension to the conversation. For example, instead of overwhelming patients with information on all possible domains and generalized statements on effectiveness, information can be focused on those specific domains that are very important to the patient and by showing the level of improvement of other patients who also valued those domains as very important.

At the same time, it is necessary for future studies to expand on our findings. For instance, by also measuring what patients actually expect on the ‘very important’ domains (measuring value-based and probabilistic expectations on the same questions). After all, a patient can find something very important but still anticipate little actual improvement on that domain. This could be reflected by the low percentages of improvement on the value-based expectations in our study.

### Do patients with higher expectation fulfillment have higher post-operative satisfaction scores?

This is the first study to investigate the relationship between value-based expectation improvement and post-operative satisfaction after SA. Even for probabilistic expectations, we only found the aforementioned study by Green et al.^3^ They found statistically significant but weak to very weak correlations between met probabilistic expectations and satisfaction (*r* = 0.17-0.37), but they did not further quantify the magnitude of that relationship. Our study elaborates on their findings, not only by quantifying the relationship but also by correcting for generalized optimism. Future studies with larger sample sizes can correct for more psychological factors like anxiety, depression, or pain catastrophizing, parallel to studies on THA and TKA.

A remarkable finding of our study is that the predicted probabilities for ‘completely improved’ expectations and ‘completely or somewhat improved’ expectations were very different at six months (see Fig. 5 *A* and *B*). Because a low percentage of ‘completely or somewhat improved’ expectations means there is little improvement at all, it would make sense that a patient would not be satisfied with that result. However, at 12 months, the difference in predicted probabilities is much smaller. Deakin et al^2^ found similar results in TKA patients: fulfilled expectations were related to satisfaction, but a low percentage of fulfillments did not exclude satisfaction. Perhaps patients adjust their frame of reference to align more with the current situation, the longer ago the surgery has been—a psychological phenomenon called resilience. It would be interesting to gain more insight into such potential response shifts in future studies.

### Limitations

Despite the study's strengths, we also need to address some clear limitations. The first limitation is that we did not measure the exact same items probabilistically as we did value-based. Assessing probabilistic expectations with the HSS would have enabled us to identify whether the ‘very important’ expectations were truly fulfilled, instead of only measuring how much patients improved on those domains. Nevertheless, since this is the first study that measured and evaluated both probabilistic and value-based expectations in the same sample, our results still deepen our understanding of how different kinds of expectations relate to satisfaction.

Second, we analyzed aTSA and rTSA patients together as a single group, but the extent to which certain expectations (eg, ROM) can be fulfilled/improved can differ between aTSA and rTSA. We also included small numbers of patients undergoing HA or revision arthroplasty. However, we expect these differences to only affect the absolute levels of fulfillment/improvement and not the relationship between fulfillment/improvement and satisfaction. Moreover, Lawrence et al^8^ did not find important differences in cumulative expectation scores and single expectations between aTSA and rTSA patients.

Finally, the pre-operative counseling (and thus pre-operative expectation management), treatment and aftercare were not standardized between the participating hospitals and surgeons. It could therefore be that expectations were managed differently between patients. The upside of this limitation is that our results might be better applicable to other settings than only the participating hospitals, as these differences exist in the real world as well. We expect that our results are also generalizable to different health-care system structures than in the Netherlands, since the expectations in our questionnaires specifically concern shoulder pain and function, rather than properties of the health-care process itself.

## Conclusion

This study shows that fulfillment of probabilistic expectations and improvement on value-based expectations is associated with post-operative satisfaction after SA. Surgeons and other health-care professionals can use these results to further customize and optimize patient education and expectation management: generalized statements about recovery can now be supplemented with statements on specific domains that are important to patients.

Future studies should elaborate on our results by measuring and comparing probabilistic and value-based expectations on the same instrument and items in 1 population, and by predicting the probability of complete expectation fulfillment/improvement on an individual level.

## Disclaimers:

Funding: This study received financial support for printing costs from the Scientific Committee of the Reinier de Graaf Hospital, Delft, the Netherlands (grant no. 621704).

Conflicts of interest: The authors, their immediate families, and any research foundation with which they are affiliated have not received any financial payments or other benefits from any commercial entity related to the subject of this article.
