# Supplementary material for: Complete fulfillment of expectations is associated with greater satisfaction after shoulder arthroplasty: results from a prospective multicenter cohort study
Source: JSES Int. 2026 Jan 29;10(3):101651. doi: 10.1016/j.jseint.2026.101651 (PMC13049639; doi:10.1016/j.jseint.2026.101651)
Supplement: Supplementary File [file mmc1.docx]

**Supplementary file**

# Table S1 – Preoperative Sunnybrook questionnaire

| Q1. Do you expect your surgery to help with pain relief?   - Not applicable, I do not have pain - No, I do not expect surgery to help with my pain - Yes, but just a little - Yes, somewhat - Yes, a lot |
| --- |
| Q2. Do you expect your surgery to increase your pain-free range of motion?   - Not applicable, I do not have restricted range - No, I do not expect surgery to increase my pain-free range of motion - Yes, but just a little - Yes, somewhat - Yes, a lot |
| Q3. Do you expect your surgery to improve your ability to carry out the normal activities of daily living?   - Not applicable, I do not have problems with activities of daily living - No, I do not expect surgery to improve my ability to carry out the normal daily activities - Yes, but just a little - Yes, somewhat - Yes, a lot |
| Q4. Do you expect your surgery to improve your ability to care for others?   - Not applicable, I do not have problems interacting and caring for others - No, I do not expect surgery to improve my ability to interact and care for others - Yes, but just a little - Yes, somewhat - Yes, a lot |
| Q5. Do you expect that following your surgery you will be able to participate in the leisure, sports, or recreational activities you did before your problem started?   - Not applicable (did not do sports or recreational activities before) - No, I do not expect surgery to improve my participation in sport/recreational activities - Yes, but not as much as before - Yes, as much as before |
| Q6. Do you expect that following your surgery the area operated upon will be back to the way it was before you began having problems there?   - No, I do not expect the area operated on to be back to the way it was before I had problems there - No, but a little improved - No, but somewhat improved - Yes, completely |

# Table S2 – Modified postoperative Sunnybrook questionnaire

| Q1. Did your surgery help with pain relief?   - Not applicable, I did not have pain - No, the surgery did not help with pain relief - Yes, but just a little - Yes, somewhat - Yes, a lot |
| --- |
| Q2. Did your surgery increase your pain-free range of motion?   - Not applicable, I did not have restricted range - No, the surgery did not increase my pain-free range of motion - Yes, but just a little - Yes, somewhat - Yes, a lot |
| Q3. Did your surgery improve your ability to carry out the normal activities of daily living?   - Not applicable, I did not have problems with activities of daily living - No, the surgery did not improve my ability to carry out the normal daily activities - Yes, but just a little - Yes, somewhat - Yes, a lot |
| Q4. Did your surgery improve your ability to care for others?   - Not applicable, I did not have problems interacting and caring for others - No, the surgery did not improve my ability to interact and care for others - Yes, but just a little - Yes, somewhat - Yes, a lot |
| Q5. After your surgery, are you able to participate in the leisure, sports, or recreational activities you did before your problem started?   - Not applicable (did not do sports or recreational activities before) - No, I cannot participate in sport/recreational activities - Yes, but not as much as before - Yes, as much as before |
| Q6. After your surgery, is the area operated upon back to the way it was before you began having problems there?   - No, the area operated on is not back to the way it was before I had problems there - No, but a little improved - No, but somewhat improved - Yes, completely |

| **How important are these expectations in the treatment for your shoulder?** | **Very important** | **Somewhat important** | **A little important** | **I do not expect this** | **This does not apply to me** |
| --- | --- | --- | --- | --- | --- |
| Relieve daytime pain (HSS1) | 1 | 2 | 3 | 4 | 5 |
| Relieve nighttime pain (HSS2) | 1 | 2 | 3 | 4 | 5 |
| Improve shoulder range of motion (HSS3) | 1 | 2 | 3 | 4 | 5 |
| Stop shoulder from dislocating (HSS4) | 1 | 2 | 3 | 4 | 5 |
| Stop shoulder from clicking (HSS5) | 1 | 2 | 3 | 4 | 5 |
| Improve ability to carry objects over 10 pounds (HSS6) | 1 | 2 | 3 | 4 | 5 |
| Improve ability to reach above shoulder level (for example, reach a high shelf) (HSS7) | 1 | 2 | 3 | 4 | 5 |
| Improve ability to reach sideways (HSS8) | 1 | 2 | 3 | 4 | 5 |
| Improve self-care (for example, wash, dress) (HSS9) | 1 | 2 | 3 | 4 | 5 |
| Be employed for monetary reimbursement (HSS10) | 1 | 2 | 3 | 4 | 5 |
| Improve psychological well-being (HSS11) | 1 | 2 | 3 | 4 | 5 |
| Improve ability to interact with others (for example, take care of someone, play with children) (HSS12) | 1 | 2 | 3 | 4 | 5 |
| Improve ability to perform daily activities (for example, daily routine, household chores) (HSS13) | 1 | 2 | 3 | 4 | 5 |
| Improve ability to drive or put on a seatbelt (HSS14) | 1 | 2 | 3 | 4 | 5 |
| Improve ability to exercise or participate in sports (HSS15) | 1 | 2 | 3 | 4 | 5 |
| Improve ability to participate in recreational activities (for example, gardening, dancing) (HSS16) | 1 | 2 | 3 | 4 | 5 |
| For shoulder to be back to the way it was before this problem started (HSS17)  HSS = Hospital for Special Surgery | 1 | 2 | 3 | 4 | 5 |

# Table S3 – Preoperative HSS questionnaire

# Table S4 – Modified postoperative HSS questionnaire

| **For these expectations, which answer fits your situation best?** | **Complete improvement** | **Much improvement** | **Moderate improvement** | **A little improvement** | **No improvement / no deterioration** | **A little deterioration** | **Moderate deterioration** | **Much deterioration** |
| --- | --- | --- | --- | --- | --- | --- | --- | --- |
| Relieve daytime pain (HSS1) | 1 | 2 | 3 | 4 | 5 | 6 | 7 | 8 |
| Relieve nighttime pain (HSS2) | 1 | 2 | 3 | 4 | 5 | 6 | 7 | 8 |
| Improve shoulder range of motion (HSS3) | 1 | 2 | 3 | 4 | 5 | 6 | 7 | 8 |
| Stop shoulder from dislocating (HSS4) | 1 | 2 | 3 | 4 | 5 | 6 | 7 | 8 |
| Stop shoulder from clicking (HSS5) | 1 | 2 | 3 | 4 | 5 | 6 | 7 | 8 |
| Improve ability to carry objects over 10 pounds (HSS6) | 1 | 2 | 3 | 4 | 5 | 6 | 7 | 8 |
| Improve ability to reach above shoulder level (for example, reach a high shelf) (HSS7) | 1 | 2 | 3 | 4 | 5 | 6 | 7 | 8 |
| Improve ability to reach sideways (HSS8) | 1 | 2 | 3 | 4 | 5 | 6 | 7 | 8 |
| Improve self-care (for example, wash, dress) (HSS9) | 1 | 2 | 3 | 4 | 5 | 6 | 7 | 8 |
| Be employed for monetary reimbursement (HSS10) | 1 | 2 | 3 | 4 | 5 | 6 | 7 | 8 |
| Improve psychological well-being (HSS11) | 1 | 2 | 3 | 4 | 5 | 6 | 7 | 8 |
| Improve ability to interact with others (for example, take care of someone, play with children) (HSS12) | 1 | 2 | 3 | 4 | 5 | 6 | 7 | 8 |
| Improve ability to perform daily activities (for example, daily routine, household chores) (HSS13) | 1 | 2 | 3 | 4 | 5 | 6 | 7 | 8 |
| Improve ability to drive or put on a seatbelt (HSS14) | 1 | 2 | 3 | 4 | 5 | 6 | 7 | 8 |
| Improve ability to exercise or participate in sports (HSS15) | 1 | 2 | 3 | 4 | 5 | 6 | 7 | 8 |
| Improve ability to participate in recreational activities (for example, gardening, dancing) (HSS16) | 1 | 2 | 3 | 4 | 5 | 6 | 7 | 8 |
| For shoulder to be back to the way it was before this problem started (HSS17) | 1 | 2 | 3 | 4 | 5 | 6 | 7 | 8 |

HSS = Hospital for Special Surgery

# Question 2 - statistical analysis

For probabilistic expectations, expectation fulfilment for each expectation was categorized as:

- ‘completely fulfilled’: if the patient postoperatively scored at least the same or higher level of improvement as preoperatively expected (exceeded expectations were included in ‘completely fulfilled’)
- ‘somewhat fulfilled’: if the postoperative score indicated improvement but less than preoperatively expected,
- ‘not at all fulfilled’: if the postoperative score indicated no improvement.

For value-based expectations, we collapsed our 8-point Likert scale into similar categories:

- ‘completely improved’ contained only the answer ‘complete improvement’,
- ‘somewhat improved’ contained ‘much improvement’, ‘moderate improvement’ and ‘a little improvement’
- ‘Not at all improved’ contained the remaining answers (including deterioration).

Subsequently, for each probabilistic expectation, the following percentages were calculated at six months and at twelve months:

$\% completely fulfilled=\frac{no. of patients with that expectation who scored ‘completely fulfilled’}{no. of patients with that expectation}$

$\% completely/somewhat fulfilled=\frac{no. of patients with that expectation who scored ‘completely or somewhat fulfilled’}{no. of patients with that expectation}$

Similar to the probabilistic expectations, we calculated the following percentages for each value-based expectation at six months and at twelve months:

$\% completely improved=\frac{\begin{aligned} no. of patients with that expectation rated 'very \mathrm{important}^{'} who scored \\ ‘completely improved' \end{aligned}}{no. of patients with that expectation as 'very important'}$

$\% completely/somewhat improved=\frac{\begin{aligned} no. of patients with that expectation as 'very \mathrm{important}^{'} who scored \\ ‘completely or somewhat improved' \end{aligned}}{no. of patients with that expectation as 'very important'}$

# Question 3 - statistical analysis

We encountered problems with two models. Firstly, the model for ‘completely improved’ value-based expectations at twelve months produced an error. After investigation, we determined the cause to be that all patients who were ‘not satisfied’ had 0% of their ‘very important’ expectations ‘completely improved’; this group had no variation. After conferring with our statistician, we adjusted the percentage ‘completely improved’ of one patient to 5%. This made sure the model could run while it simultaneously did not impact the magnitude of any possible association. Secondly, the model for ‘completely fulfilled’ probabilistic expectations at twelve months did run, but the Lipsitz goodness of fit test indicated that the model did not fit our data well. Possible adjustments to the model, like adding an interaction term or using splines, would lead to overfitting and was thus considered not statistically sound. We therefore evaluated the relationship between ‘completely fulfilled’ probabilistic expectations and satisfaction at twelve months in a qualitative manner.

# Figure S1 (appendix) – Probabilistic expectation fulfillment at each time point, per category


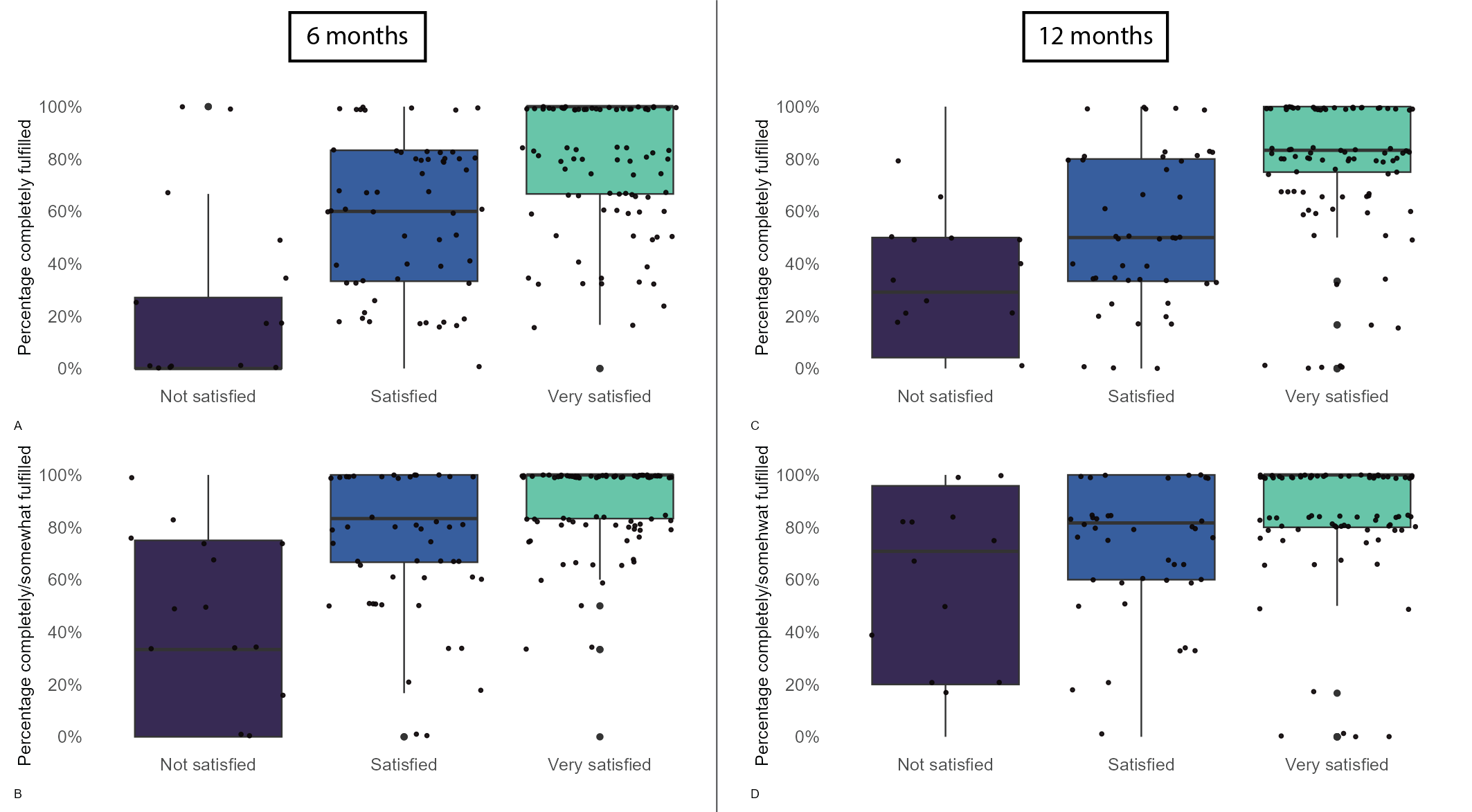


Figure S1A-D. Boxplots for probabilistic expectations showing per satisfaction level the median and IQR of (A) percentage ‘completely fulfilled’ expectations at 6 months, (B) percentage ‘completely or somewhat fulfilled’ expectations at 6 months, (C) percentage ‘completely fulfilled’ expectations at 12 months, and (D) percentage ‘completely or somewhat fulfilled’ expectations at 12 months.

# Figure S2 (appendix) – Value-based expectation improvement at each time point, per category


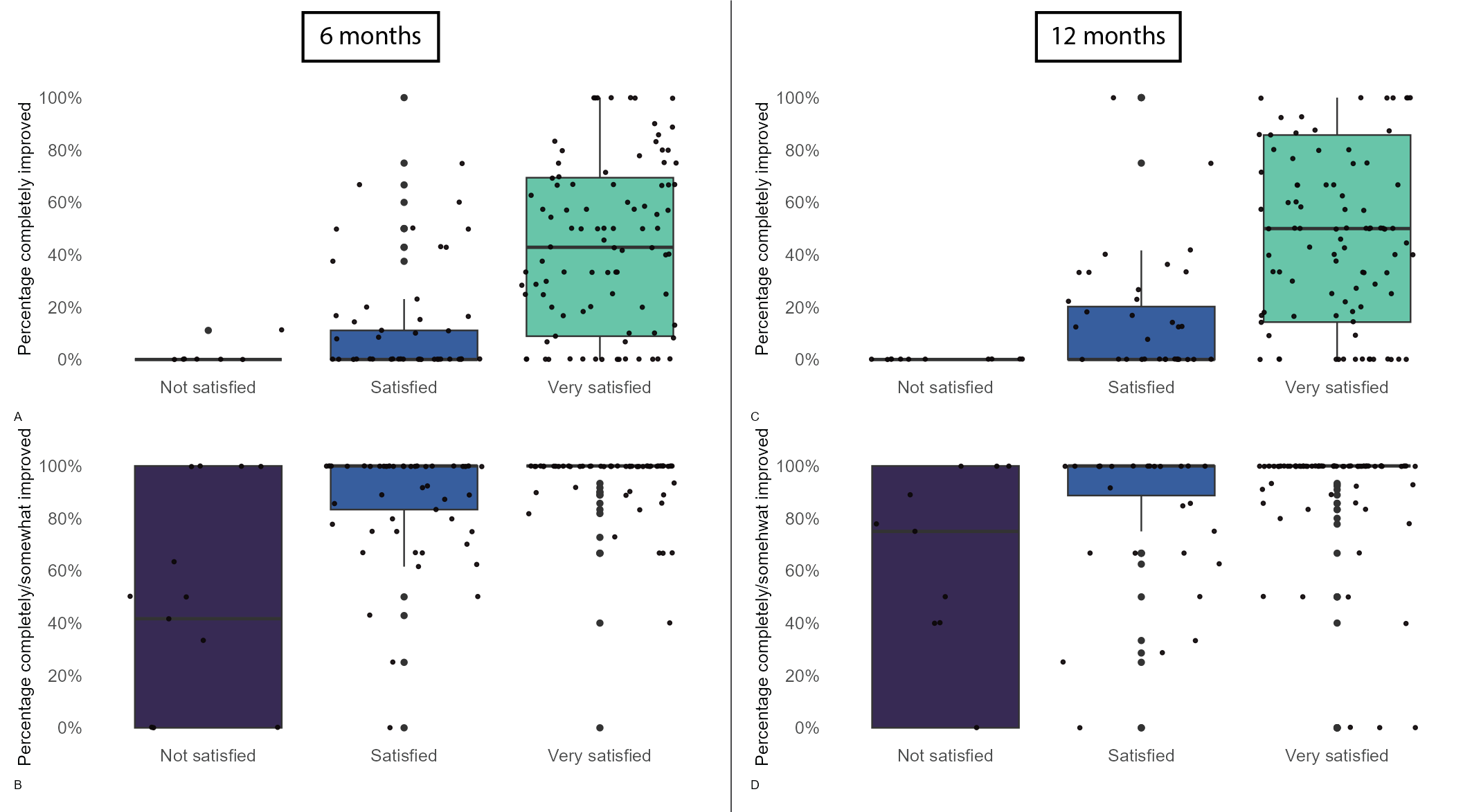


Figure S2A-D. Boxplots for value-based expectations showing per satisfaction level the median and IQR of (A) percentage ‘completely improved’ expectations at 6 months, (B) percentage ‘completely or somewhat improved’ expectations at 6 months, (C) percentage ‘completely improved’ expectations at 12 months, and (D) percentage ‘completely or somewhat improved’ expectations at 12 months.

# Table S5 (appendix) - Baseline probabilistic expectations

| **Table S5. Baseline probabilistic expectations (Sunnybrook questionnaire)** | |
| --- | --- |
| **Questionnaire item** | **Entire sample**  **(N = 230)** |
| *Pain relief (Q.1)* | |
| Yes, a lot | 163 (70.9%) |
| Yes, somewhat | 47 (20.4%) |
| Yes, but just a little | 1 (0.4%) |
| Does not apply | 4 (1.7%) |
| Missing | 15 (6.5%) |
| *Range of motion (Q.2)* | |
| Yes, a lot | 117 (50.9%) |
| Yes, somewhat | 80 (34.8%) |
| Yes, but just a little | 10 (4.3%) |
| Do not expect this | 5 (2.2%) |
| Does not apply | 3 (1.3%) |
| Missing | 15 (6.5%) |
| *Ability to carry out normal ADL (Q.3)* | |
| Yes, a lot | 113 (49.1%) |
| Yes, somewhat | 78 (33.9%) |
| Yes, but just a little | 15 (6.5%) |
| Do not expect this | 4 (1.7%) |
| Does not apply | 5 (2.2%) |
| Missing | 15 (6.5%) |
| *Ability to care for others (Q.4)* | |
| Yes, a lot | 59 (25.7%) |
| Yes, somewhat | 66 (28.7%) |
| Yes, but just a little | 24 (10.4%) |
| Do not expect this | 7 (3.0%) |
| Does not apply | 59 (25.7%) |
| Missing | 15 (6.5%) |
| *Participate in leisure, sports or recreational activities (Q.5)* | |
| Yes, as much as before | 49 (21.3%) |
| Yes, but not as much as before | 105 (45.7%) |
| Do not expect this | 11 (4.8%) |
| Does not apply | 49 (21.3%) |
| Missing | 16 (7.0%) |
| *Shoulder back to the way it was (Q.6)* | |
| Yes, completely | 78 (33.9%) |
| No, but somewhat improved | 115 (50.0%) |
| No, but a little improved | 3 (1.3%) |
| Do not expect this | 18 (7.8%) |
| Missing | 16 (7.0%) |
|  |  |

# Table S6 (appendix) - Baseline value-based expectations

| **Table S6. Baseline value-based expectations (HSS questionnaire)** | |
| --- | --- |
| **Questionnaire item** | **Entire sample**  **(N = 230)** |
| *Relieve daytime pain (HSS 1)* | |
| Very important | 132 (57.4%) |
| Somewhat important | 73 (31.7%) |
| A little important | 8 (3.5%) |
| Does not apply | 3 (1.3%) |
| Missing | 14 (6.1%) |
| *Relieve nighttime pain (HSS 2)* | |
| Very important | 142 (61.7%) |
| Somewhat important | 55 (23.9%) |
| A little important | 9 (3.9%) |
| Do not expect this | 1 (0.4%) |
| Does not apply | 8 (3.5%) |
| Missing | 15 (6.5%) |
| *Improve shoulder ROM (HSS 3)* | |
| Very important | 134 (58.3%) |
| Somewhat important | 69 (30.0%) |
| A little important | 9 (3.9%) |
| Do not expect this | 4 (1.7%) |
| Missing | 14 (6.1%) |
| *Stop shoulder from dislocating (HSS 4)* | |
| Very important | 8 (3.5%) |
| Somewhat important | 5 (2.2%) |
| A little important | 4 (1.7%) |
| Do not expect this | 4 (1.7%) |
| Does not apply | 194 (84.3%) |
| Missing | 15 (6.5%) |
| *Stop shoulder from clicking (HSS 5)* | |
| Very important | 33 (14.3%) |
| Somewhat important | 44 (19.1%) |
| A little important | 30 (13.0%) |
| Do not expect this | 3 (1.3%) |
| Does not apply | 105 (45.7%) |
| Missing | 15 (6.5%) |
| *Carry objects over 10 pounds (HSS 6)* | |
| Very important | 56 (24.3%) |
| Somewhat important | 65 (28.3%) |
| A little important | 53 (23.0%) |
| Do not expect this | 21 (9.1%) |
| Does not apply | 21 (9.1%) |
| Missing | 14 (6.1%) |
| *Reach above shoulder level (HSS 7)* | |
| Very important | 99 (43.0%) |
| Somewhat important | 79 (34.3%) |
| A little important | 21 (9.1%) |
| Do not expect this | 12 (5.2%) |
| Does not apply | 5 (2.2%) |
| Missing | 14 (6.1%) |
| *Reach sideways (HSS 8)* | |
| Very important | 89 (38.7%) |
| Somewhat important | 93 (40.4%) |
| A little important | 25 (10.9%) |
| Do not expect this | 8 (3.5%) |
| Does not apply | 1 (0.4%) |
| Missing | 14 (6.1%) |
| *Improve self-care (HSS 9)* | |
| Very important | 110 (47.8%) |
| Somewhat important | 63 (27.4%) |
| A little important | 16 (7.0%) |
| Do not expect this | 2 (0.9%) |
| Does not apply | 25 (10.9%) |
| Missing | 14 (6.1%) |
| *Paid work (HSS 10)* | |
| Very important | 20 (8.7%) |
| Somewhat important | 12 (5.2%) |
| A little important | 9 (3.9%) |
| Do not expect this | 6 (2.6%) |
| Does not apply | 169 (73.5%) |
| Missing | 14 (6.1%) |
| *Psychological well-being (HSS 11)* | |
| Very important | 45 (19.6%) |
| Somewhat important | 54 (23.5%) |
| A little important | 29 (12.6%) |
| Do not expect this | 9 (3.9%) |
| Does not apply | 79 (34.3%) |
| Missing | 14 (6.1%) |
| *Interact with others (HSS 12)* | |
| Very important | 52 (22.6%) |
| Somewhat important | 76 (33.0%) |
| A little important | 25 (10.9%) |
| Do not expect this | 9 (3.9%) |
| Does not apply | 53 (23.0%) |
| Missing | 15 (6.5%) |
| *Perform daily activities (HSS 13)* | |
| Very important | 100 (43.5%) |
| Somewhat important | 86 (37.4%) |
| A little important | 23 (10.0%) |
| Do not expect this | 1 (0.4%) |
| Does not apply | 5 (2.2%) |
| Missing | 15 (6.5%) |
| *Drive or put on a seatbelt (HSS 14)* | |
| Very important | 76 (33.0%) |
| Somewhat important | 63 (27.4%) |
| A little important | 35 (15.2%) |
| Do not expect this | 4 (1.7%) |
| Does not apply | 37 (16.1%) |
| Missing | 15 (6.5%) |
| *Exercise/participate in sports (HSS 15)* | |
| Very important | 65 (28.3%) |
| Somewhat important | 75 (32.6%) |
| A little important | 43 (18.7%) |
| Do not expect this | 9 (3.9%) |
| Does not apply | 23 (10.0%) |
| Missing | 15 (6.5%) |
| *Recreational activities (HSS 16)* | |
| Very important | 56 (24.3%) |
| Somewhat important | 68 (29.6%) |
| A little important | 40 (17.4%) |
| Do not expect this | 14 (6.1%) |
| Does not apply | 37 (16.1%) |
| Missing | 15 (6.5%) |
| *Shoulder back to the way it was (HSS 17)* | |
| Very important | 104 (45.2%) |
| Somewhat important | 75 (32.6%) |
| A little important | 2 (0.9%) |
| Do not expect this | 32 (13.9%) |
| Does not apply | 2 (0.9%) |
| Missing | 15 (6.5%) |
|  |  |

HSS = Hospital for Special Surgery

# Table S7 (appendix) – Postoperative probabilistic expectation fulfillment

| **Table S7. Postoperative probabilistic expectation fulfillment (Sunnybrook questionnaire)** | | |
| --- | --- | --- |
| **Questionnaire item** | **6 Months** | **12 Months** |
| *Pain relief (Q.1)* | | |
| Completely fulfilled | 157 (85.3%) | 154 (86.5%) |
| Somewhat fulfilled | 16 (8.7%) | 16 (9%) |
| Not at all fulfilled | 11 (6%) | 8 (4.5%) |
| *Range of motion (Q.2)* | | |
| Completely fulfilled | 136 (74.7%) | 134 (75.7%) |
| Somewhat fulfilled | 24 (13.2%) | 30 (16.9%) |
| Not at all fulfilled | 22 (12.1%) | 13 (7.3%) |
| *Ability to carry out normal ADL (Q.3)* | | |
| Completely fulfilled | 127 (70.6%) | 127 (73.8%) |
| Somewhat fulfilled | 40 (22.2%) | 31 (18%) |
| Not at all fulfilled | 13 (7.2%) | 14 (8.1%) |
| *Ability to care for others (Q.4)* | | |
| Completely fulfilled | 75 (70.1%) | 77 (72%) |
| Somewhat fulfilled | 21 (19.6%) | 20 (18.7%) |
| Not at all fulfilled | 11 (10.3%) | 10 (9.3%) |
| *Participate in leisure, sports or recreational activities (Q.5)* | | |
| Completely fulfilled | 82 (64.6%) | 88 (73.9%) |
| Somewhat fulfilled | 23 (18.1%) | 15 (12.6%) |
| Not at all fulfilled | 22 (17.3%) | 16 (13.4%) |
| *Shoulder back to the way it was (Q.6)* | | |
| Completely fulfilled | 115 (65.7%) | 113 (73.4%) |
| Somewhat fulfilled | 27 (15.4%) | 16 (10.4%) |
| Not at all fulfilled | 33 (18.9%) | 25 (16.2%) |
|  |  |  |

# Table S8 (appendix) – Postoperative value-based expectation improvement

| **Table S8. Postoperative value-based expectation improvement**  **(HSS questionnaire)** | | |
| --- | --- | --- |
| **Questionnaire item** | **6 Months** | **12 Months** |
| *Relieve daytime pain (HSS 1)* | | |
| Completely improved | 49 (37.1%) | 49 (37.1%) |
| Somewhat improved | 65 (49.2%) | 64 (48.5%) |
| Not at all improved | 18 (13.6%) | 19 (14.4%) |
| *Relieve nighttime pain (HSS 2)* | | |
| Completely improved | 46 (32.4%) | 59 (41.5%) |
| Somewhat improved | 81 (57%) | 65 (45.8%) |
| Not at all improved | 15 (10.6%) | 18 (12.7%) |
| *Improve shoulder ROM (HSS 3)* | | |
| Completely improved | 26 (19.4%) | 34 (25.4%) |
| Somewhat improved | 90 (67.2%) | 79 (59%) |
| Not at all improved | 18 (13.4%) | 21 (15.7%) |
| *Stop shoulder from dislocating (HSS 4)* | | |
| Completely improved | 6 (75%) | 5 (62.5%) |
| Somewhat improved | 1 (12.5%) | 1 (12.5%) |
| Not at all improved | 1 (12.5%) | 2 (25%) |
| *Stop shoulder from clicking (HSS 5)* | | |
| Completely improved | 14 (42.4%) | 16 (48.5%) |
| Somewhat improved | 14 (42.4%) | 8 (24.2%) |
| Not at all improved | 5 (15.2%) | 9 (27.3%) |
| *Carry objects over 10 pounds (HSS 6)* | | |
| Completely improved | 9 (16.1%) | 18 (32.1%) |
| Somewhat improved | 38 (67.9%) | 29 (51.8%) |
| Not at all improved | 9 (16.1%) | 9 (16.1%) |
| *Reach above shoulder level (HSS 7)* | | |
| Completely improved | 27 (27.3%) | 41 (41.4%) |
| Somewhat improved | 58 (58.6%) | 45 (45.5%) |
| Not at all improved | 14 (14.1%) | 13 (13.1%) |
| *Reach sideways (HSS 8)* | | |
| Completely improved | 23 (25.8%) | 35 (39.3%) |
| Somewhat improved | 54 (60.7%) | 39 (43.8%) |
| Not at all improved | 12 (13.5%) | 15 (16.9%) |
| *Improve self-care (HSS 9)* | | |
| Completely improved | 53 (48.2%) | 48 (43.6%) |
| Somewhat improved | 44 (40%) | 44 (40%) |
| Not at all improved | 13 (11.8%) | 18 (16.4%) |
| *Paid work (HSS 10)* | | |
| Completely improved | 2 (10%) | 3 (15%) |
| Somewhat improved | 7 (35%) | 8 (40%) |
| Not at all improved | 11 (55%) | 9 (45%) |
| *Psychological well-being (HSS 11)* | | |
| Completely improved | 9 (20%) | 12 (26.7%) |
| Somewhat improved | 26 (57.8%) | 18 (40%) |
| Not at all improved | 10 (22.2%) | 15 (33.3%) |
| *Interact with others (HSS 12)* | | |
| Completely improved | 13 (25%) | 15 (28.8%) |
| Somewhat improved | 30 (57.7%) | 29 (55.8%) |
| Not at all improved | 9 (17.3%) | 8 (15.4%) |
| *Perform daily activities (HSS 13)* | | |
| Completely improved | 20 (20%) | 29 (29%) |
| Somewhat improved | 67 (67%) | 49 (49%) |
| Not at all improved | 13 (13%) | 22 (22%) |
| *Drive or put on a seatbelt (HSS 14)* | | |
| Completely improved | 24 (31.6%) | 28 (36.8%) |
| Somewhat improved | 39 (51.3%) | 31 (40.8%) |
| Not at all improved | 13 (17.1%) | 17 (22.4%) |
| *Exercise/participate in sports (HSS 15)* | | |
| Completely improved | 14 (21.5%) | 15 (23.1%) |
| Somewhat improved | 34 (52.3%) | 35 (53.8%) |
| Not at all improved | 17 (26.2%) | 15 (23.1%) |
| *Recreational activities (HSS 16)* | | |
| Completely improved | 11 (19.6%) | 18 (32.1%) |
| Somewhat improved | 31 (55.4%) | 29 (51.8%) |
| Not at all improved | 14 (25%) | 9 (16.1%) |
| *Shoulder back to the way it was (HSS 17)* | | |
| Completely improved | 18 (17.3%) | 24 (23.1%) |
| Somewhat improved | 69 (66.3%) | 61 (58.7%) |
| Not at all improved | 17 (16.3%) | 19 (18.3%) |
|  |  |  |

HSS = Hospital for Special Surgery
